# The development of self skills in an authentic learning environment: A qualitative study

**DOI:** 10.4102/curationis.v45i1.2198

**Published:** 2022-01-18

**Authors:** Gugu Ndawo

**Affiliations:** 1 Department of Nursing, Faculty Health Sciences, University of Johannesburg, Johannesburg, South Africa

**Keywords:** authentic learning, nursing education, self, self-awareness, self skills

## Abstract

**Background:**

The contemporary healthcare environment is an authentic, demanding, challenging and ever-changing environment that requires learners to possess good self skills when they need to engage in meaningful, critical discourse in order to solve authentic problems. However, nurse educators assume that learners already have well-developed self skills at the commencement of their nursing training and as a result do not explicitly teach and develop such skills in the learners.

**Objectives:**

The objectives of this research were to explore and describe nurse educators’ views on how learners’ self skills can be developed within an authentic learning (AL) environment, and to formulate recommendations based on the findings.

**Method:**

A qualitative and contextual research design was used to seek rich, in-depth data from 20 nurse educators who were purposively sampled. Semi-structured individual interviews were conducted, and the data were analysed using Miles, Huberman and Saldaña method.

**Results:**

The three themes that emerged were that nurse educators should (1) ensure an AL environment that promotes self skills, (2) engage learners in activities that will consciously evoke authentic self and (3) evaluate the developed self skills and metacognition.

**Conclusion:**

By developing good self skills, learners should be able to deliver quality patient care, find solutions to complex problems and handle cognitive complexity and authentic conditions whilst creating their own identity.

## Introduction

The current healthcare environment is a demanding, challenging and ever-changing interplay of inevitable key changes that take place nationally and globally on political, economic, social, demographic, competitive and technological levels. These changes call for nursing education institutions to produce highly skilled healthcare graduates who are able to meet the unrelenting demands of the unpredictable healthcare environment of the 21st century. To succeed, it is essential that the training develops crucial self skills that will assist graduates to continually adapt to the healthcare uncertainties (Dyson 2018). However, for learners to be able to successfully adapt to changing circumstances, they need to learn how to obtain new skills within the shortest period because these inevitable key changes occur rapidly. They therefore need to acquire the skills that will help them to gain new knowledge throughout their professional lives (Amirkhanova et al. 2017). Whilst developing such skills, it is also important that learners engage in authentic learning (AL) activities that will develop their self skills so that they can survive and thrive within an AL environment.

An AL environment is a complex, challenging real-life or virtual setting in which learners engage in activities that mimic the real professional world where they are expected to simulate their acquired knowledge (Vereijken et al. 2019). To enable learners to develop and acquire good self skills, such an environment must consist of nine elements, namely the authentic context, authentic tasks, access to experts, multiple roles and perspectives, collaborative construction of knowledge, reflection, articulation, coaching and scaffolding and authentic assessments (Herrington, Reeves & Oliver 2010 in Huang-Saad et al. 2021). In this setting, learners can develop their self skills by engaging with more experienced individuals in a quest to develop solutions for patients’ authentic problems. To realise this goal, learners must fully engage physically and virtually in AL activities and assessments such as problem-based learning, real-life case studies, debates and arguments, critical discourse and conversations with experts, formulating research papers within communities of learners, creating videos, writing reflective eJournals and ePortfolios and self and peer evaluations.

Nurse educators need to take cognisance that such AL activities can be extremely challenging and frustrating for novice learners, and as a result, the learners often face difficulty in acquiring self skills as well as higher-order thinking skills on their own without scaffolding. Irrespective of the challenges of AL, nurse educators should aspire to develop these skills by immersing learners in real-world, ill-defined, challenging, complex problems, and authentic assessments (Cañas, Reiska & Möllits 2017).

As a learner-centred approach, AL is anchored in philosophical and theoretical foundations that include participative, experiential, andragogical, motivational, humanistic, activity-based and constructivist, as well as situation- and problem-based learning approaches. By the very nature of the authentic problems within these foundations, learners are compelled to interact, communicate and collaborate meaningfully and effectively with many more-abled individuals such as global experts, communities of practice, educators, peers and real communities (Koretsky et al. 2019) whilst using digital technology as a cognitive tool (Drew 2019). In such situations, learners should have good self skills, good communication skills, positive face-to-face interactions as well as good netiquette skills to enable respectful, meaningful interactions and engagements. Good self skills enable the learner to effectively manage the self and deal with the intense emotional engagement that entails both constructive and unstructured interactions within an AL environment (Darling-Hammond et al. 2019).

The self skills are the personal abilities that learners develop and use when engaged with more-abled others within an AL environment, and they include self-awareness, self-dialogue, self-inquiry, self-reflection and metacognition. The first question that arises in this context is who is self within such an environment? Priest defined the self as ‘an individual that is conscious of the individual that it is whilst being conscious that it is the individual it is conscious of’ (1991:163). The learner becomes a self who must be conscious of their engagement in any AL environment in pursuit of developing good self skills. Thus, the question of ‘Who am I?’ is pertinent and demands that each learner reflects upon and finds answers for meaningful AL engagement and personal growth, development and change. According to Benn (2018), the question should be explored in detail to include one’s gender, ethnic, religious, cultural, national and professional self. All these categories make up the self that will ultimately be utilised as a tool to bring about change in others including the patients. The self in AL is underpinned by humanistic theories. According to Rogers 1951 in Nelson, Groom and Potrac (2016), the interactions with the environment and particularly appraisal interactions with others result in the formation and development of self. Thus, through social interaction, there is an emergence of ‘I’ and ‘me’ in the self’s conceptual understanding. Educators who adopt a humanist perspective recognise the importance of relationships between learners and themselves, and they seek to foster meaningful connections. They understand that learners are intrinsically driven by a tendency to self-actualise (Ebert, Levett-Jones & Jones 2019).

### Problem statement

The purpose of higher education is to produce a well-rounded graduate who is a future change agent (Frank & Stanszus 2019) with intellectual capital as well as good self skills (Mydin & Amran 2019). At the same time, the higher-education institutions are pressured to meet the demands of external metrics that result in a performative culture that is immensely assessment driven, and appraisal and goal oriented. Such a culture supersedes quality education resulting in the evasion of meaningful AL academic risks that develop learner’s independent self skills. There is also an assumption by nurse educators that learners are aware of self as a cognitive agent at the commencement of the programme, but the reality is that the majority of learners lack this skill (Nørgård, Toft-Nielsen & Whitton 2017). A lack of self skills means that the learner will have difficulty in consciously opening themselves up to learning, and thus they do not develop good communication and interpersonal skills or deep and logical reasoning that are needed to engage with patients’ problems. Learners are challenged to respectfully and meaningfully engage with the patients and their families, friends and experts, and advocate for their patients within the health service (Benn 2018). An exclusion of the self in the provision of nursing care is a recipe for failure for the patients as during a pandemic such as the coronavirus disease 2019 (COVID-19), there is decreased social support for many patients because of the enforced isolation. Learners need to be present as they, by default, assume the role of surrogate ‘family’ for the isolated, secluded patient, and as a result must unreservedly give their authentic self in caring for them in a holistic, compassionate and comprehensive manner (Hossain & Clatty 2021). To expect learners to give their self to patients is unjust when such skills are not being continually nurtured and developed. Thus, from this argument, the following question emerged: How can self skills be developed within an AL environment?

### Research aim

The aim of this research is to provide the reader with a deeper understanding of how nurse educators can develop learners’ self skills within an AL environment.

The study explored and described nurse educators’ views on the development of learners’ self skills within an AL environment with a view to formulate recommendations based on the findings.

## Research design and method

The research design was qualitative and contextual in nature, and was used to seek rich, in-depth data by exploring and describing the views of nurse educators (Gray, Grove & Sutherland 2021). Through the use of this design, the nurse educators were encouraged to share their views on their everyday, real-life situations on how they thought learners’ self skills could be developed within an AL environment. The study was contextual in nature as it was conducted in settings in which nurse educators felt comfortable to share their views (Gray et al. 2021). Their familiar settings encouraged free articulation of nurse educators’ views and assisted the researcher in describing and attaching meaning to the findings within these contexts.

### Research setting

The study was conducted in the department of nursing at the University of Johannesburg, where the researcher taught second-year undergraduate learners, supervised master’s students and worked with participants as colleagues. At the time of the study, the department offered the following programmes: a 4-year undergraduate bachelor’s degree, a 3-year post-basic bachelor’s degree, post-basic diplomas in nursing science such as nursing education, nursing administration, community, critical care, advanced midwifery and primary health, as well as master’s and doctoral degrees. The department thus catered for undergraduate, post-basic and postgraduate learners.

### Bracketing

The study was descriptive and not interpretive in nature, and thus, self-reflection was maintained through the use of a reflective diary from the commencement of the research once the phenomenon of interest was chosen and was kept throughout the research process. This diary was shared with two colleagues who assisted with peer debriefing where meaningful dialogues were held with them. These dialogues assisted the researcher in the process of disengaging self by providing provocative discussions on the research, collected data and findings using the researcher’s reflective diary. Neither of these two colleagues took part in nor assisted with the interviews, thus, were not involved in the research. During the interviews, the researcher did not bring any of her preconceived ideas as one question was posed to all participants, and only probed and paraphrased their responses as well as sought understanding through asking a question: ‘What does … mean?’ Therefore, no questions relating to the researcher’s own experiences, knowledge or preconceived ideas were posed (Kim et al. 2020; Patton 2020). The researcher also took field notes, which included methodological, theoretical, observational and personal notes (in the form of the reflective diary). These notes were shared with an independent coder during data analysis stage. Follow-up interviews with participants were conducted to confirm the accuracy of the data as their true, unchanged, unbiased views.

### Population and sampling strategy

A sample of 17 female and three male nurse educators consented to participate in the study. Consequently, rich, in-depth data were collected until no new information emerged (Gray et al. 2021). Thirteen (13) interviews led to data saturation, and the remaining seven (7) were conducted to confirm the data. The ages of the participants ranged from 37 years to 65 years, and they each had between three (*n* = 3) and 12 years of teaching experience in a higher education institution. The sample participants consisted of predominantly black people (*n* = 16), with the remaining being white people (*n* = 2), Asian people (*n* = 1) and mixed race people (*n* = 1) (see Table 1). Their highest qualification was Master’s degrees (*n* = 15) and doctoral studies (*n* = 5). A non-probability, purposive sampling method was used guided by the following inclusion criteria: the participants (1) had to have a nurse educator qualification and be registered with the South African Nursing Council (SANC) in terms of regulation 118 of 1987, (2) had to be employed as full-time nurse educators, (3) had to have taught for 3 years or more at a university and (4) had to be involved in the facilitation of learning in learner nurses registered in the 4-year Baccalaureus Curationis (BCur) degree programme leading to registration as a nurse (general, psychiatric and community) and midwife as stipulated by SANC in terms of regulation 425 of 1985 (as amended). The purposive sampling of these participants was also based on the programme’s expectation of the development of self skills in learners, and the participants could thus provide rich data.

**TABLE 1:** The sample: nurse educators’ demographic information.

Variable	Number
Total participants	20
Teaching experience (years)	3–12
**Highest qualification**	
Master’s	15
Doctoral studies	5
**Race**	
Black people	16
White people	2
Asian people	1
Mixed race people	1
**Gender**	
Female	17
Male	3
Age (years)	37–65
**Level of appointment**	
Lecturer	15
Senior lecturer	5

### Data collection

A total of 20 semi-structured individual interviews were conducted in English by the researcher between June 2015 and February 2016 in comfortable, natural settings chosen by the participants, which included the nursing boardroom, their homes and offices at the university. The researcher used the semi-structured individual interviews as a data collection method because they allowed flexibility regarding scope and depth during the discovery of the views of nurse educators on how self skills can be developed in learners within an AL environment (Gray et al. 2021). Furthermore, the participants chose their own dates, times and venues to ensure availability, flexibility and accessibility therefore ensuring that the interviews did not impact on their teaching, learning and assessment activities. Interviews were thus conducted during their free teaching time slots and personal day off. To guarantee accurate data capturing and transcription, the participants consented to the use of an audiotape recorder. The question that was posed to participants was: How can self skills be developed within an AL environment? Deeper probing questions, critical listening, reflecting, clarification, rephrasing and recapping were used to collect and clarify data from the participants (Murphy & Dillon 2015). Each interview lasted 45 min to 60 min depending on the responses of each participant. The collected data were enriched with field notes that included all the verbal and non-verbal communication dynamics and accounts of the things seen, heard, thought of and experienced during the interviews and the reflections on the data.

### Data analysis

The collected data were transcribed verbatim and analysed manually and independently using Miles, Huberman and Saldaña’s (2020) matrix-building method. Data analysis using matrices is a critical and challenging task that requires time, patience, precision and rigour as the quality of the conclusions drawn from a display is influenced by the data that is entered into the matrices. Therefore, the researcher engaged in complex mental processes, reasoning strategies, critical thinking and analysis during this stage. The processes of data condensation, data display in matrices and drawing and validating conclusions were followed as suggested by Miles et al. (2020). The choice of using rows and headings for data display was made. The data were repeatedly read whilst concentrating on similar patterns, thoughts and feelings to arrive at genuine responses. The identified patterns were then selected and grouped together to derive meaningful themes from nurse educators’ views on how self skills can be developed within an AL environment.

The data, including field notes, were then condensed and displayed in dense matrices whilst a record of selection, condensing and revision of entries into matrices was kept to demonstrate credibility of findings. Within matrices, the cell entries were made thicker so that more data were used to derive richer, meaningful themes. The researcher chose paraphrases supported by direct verbatim quotations instead of direct statements, general summary judgements or ratings for cell entries. This was followed by the use of codes to easily identify key information. A record of the agreement amongst participants was kept as the ‘decision rule’ in selecting data chunks for entries. Data entering process in the matrices was kept open as going back to the entered data was important for constant analysis and revision. Lastly, a meeting was later held between the independent coder and the researcher to validate the accuracy of the independently analysed findings as well as to ensure a collective review of the matrices displayed. Transcripts and field notes were used to verify the procedural adequacy of the built matrices. A completed, verified matrix was then agreed on and displayed. The minor disagreements on the matrices that arose were debated and resolved by presentation of the individual record of selection, condensing and revision of entries into matrices, the decision rule and paraphrases supported by direct verbatim quotations.

The independent coder was chosen because they held a PhD and had 21 years of experience in qualitative matrix-building data analysis. Seven (7) follow-up interviews with participants whose interviews were of interest were conducted to verify the accuracy of the transcripts and analysed findings as true accounts of their views. These interviews were arbitrarily conducted in no particular order.

### Trustworthiness

Trustworthiness was attained by attending to credibility, transferability, dependability and confirmability (Lincoln & Guba 1985). In order to ensure the credibility and confirmability of the research, the strategies of prolonged engagement, peer debriefing, triangulation and member checking were applied. Rapport with participants was built by spending time with them prior to data collection where the study information was shared, their questions concerning the research were truthfully answered and their concerns were heard. Peer debriefing was done through constantly engaging in meaningful dialogues regarding the research with two colleagues who were not part of the research. Such dialogues assisted the researcher with bracketing and ensuring credibility of the research findings. Triangulation was achieved by use of interviews, field notes, facilitative communication clarification techniques and an independent coder. Member checking was done by verifying the accuracy of transcripts and themes with seven nurse educators as true accounts of their views on how self skills in learners can be developed within an AL environment, which also assisted in ensuring credibility of the research findings. A confirmability audit trail, which included transcripts, data analysis records, decision rule and paraphrases as well as a reflective diary were kept (Gray et al. 2021), and were later given to the two colleagues and the independent coder to assist with self-detachment through thought-provoking discussions during the data analysis stage. To enhance the study’s transferability, an in-depth description of the research methodology, nominated samples and direct statements from participants and verbatim quotes were provided. The dependability was enhanced through in-depth description of the methodology used, an inquiry audit and a code-recode process of the data analysis.

## Findings

The three themes that emerged during the data analysis revealed that for nurse educators to develop good self skills in learners within an AL environment, they need to (1) ensure an AL environment that promotes such skills, (2) engage learners in activities that will consciously evoke authentic self and (3) evaluate the developed self skills and metacognition. The findings and field notes are to follow. There were 20 nurse educators each with a number ranging from nurse educator 1 to 20.

### Theme 1: Ensuring an authentic learning environment that promotes self skills

According to the participants, for self skills to be effectively developed, an emotional and psychologically safe environment conducive to AL must be created. In such an environment, learners are enabled to take risks as well as reflect on who they are without fear of being scorned, mocked or teased. Most participants felt that to be in an AL situation was much harder than being in other learning situations, and thus the environment must suit the learning approach. The following quotes support this view:

‘No student will bare their souls and engage in full self skills development unless the learning environment is emotionally and psychologically supportive [*pointing at herself*].’ (Participant 17, senior lecturer, 28 January 2016)

‘Authentic learning is very challenging and … scary … and feeling safe in this environment will allow students freedom to explore the self, to take risks, to trust and respect other people.’ (Participant 3, senior lecturer, 18 July 2015)

‘… authentic learning allows students to take risks, challengingly so … [*pause*] … to make mistakes, as well as find themselves, no one can solve problems out of “themselves” [*indicating inverted commas around themselves*].’ (Participant 4, lecturer, 08 August 2015)

### Theme 2: Engage learners in activities that will consciously evoke authentic self

The participants mentioned that when learners are engaged in AL tasks, they need to be consciously enabled to think about themselves. The mentioned activities included self-awareness, self-dialogue, self-inquiry and self-reflection – these were viewed as indispensable in the construction of the authentic self. The participants felt that the engagement in real-life situations such as community-based research developed learners’ authentic self. The following quotes underline this theme:

‘We should engage students in authentic tasks that will compel them to be self-aware … [*stressing the point*] which will develop other self skills like self-reflection and others and then developing the true self.’ (Participant 10, lecturer, 04 September 2015)

‘… when students are engaged in research especially community-based research, that is when real self is truly developed … students start to look at life differently from what they have and the problems people in the community deal with … this develops the self that is genuine and real … [*clenching fist*].’ (Participant #10, lecturer, 04 September 2015)

‘… engage students in activities of self-inquiry in order to self-discover [*seemingly pleased*].’ (Participant 8, lecturer, 25 August 2015)

### Theme 3: Evaluation of developed self skills and metacognition

Eighteen participants stated that well-developed self skills enabled development of metacognition, which resulted in lifelong learning. According to them, learners with well-developed self skills and metacognition were able to effectively solve complex problems. It also emerged from the interviews that the developed metacognition as well as self skills needed to be explicitly evaluated. The following three quotes exemplify the views of the majority of nurse educators:

‘… metacognition is developing and a lifelong learner when a learner, as self … [*thinking*] … thinks about their own thinking … [*smiling, pointing at self*] … that [*metacognition*] needs to be evaluated.’ (Participant 6, lecturer, 14 August 2015)

‘… we learned in nursing education that with metacognition you are able to solve gruelling, difficult problems [*clenching teeth*].’ (Participant 19, senior lecturer, 10 February 2016)

‘When we, as nurse educators think that we have developed such skills in students, we must evaluate them, otherwise we are not being explicit and honest with our “selves” [*indicating inverted commas around selves*].’ (Participant 4, lecturer, 08 August 2015)

## Discussion

The findings of this study provide sound support for the argument that developed self skills in learners are imperative if they are to make rational decisions and find unique solutions for problems within an AL environment. The findings are discussed below within the framework of the relevant literature.

### Theme 1: Ensuring an authentic learning environment that promotes self skills

The participants stated that an emotionally and psychologically safe AL environment develops good proper self skills that usually lead to an improved sense of self-awareness in learners. In such an environment, the learner is exposed to questioning that requires deep, diverse thinking, critical discourse, sharing, being creative and innovative, thereby building autonomy that improves self-esteem and is desirable for sharing multiple perspectives in solving an authentic problem. Learners must be consciously assisted to create such an environment for themselves and by themselves to enable them to freely and openly develop self skills. Within a psychologically safe AL environment, the learner’s inner potentiality is set out, and they are thus enabled to self-actualise (Javadi & Tahmasbi 2019). All participants believed that a safe AL environment enabled learners to explore the self unreservedly. Reassurance, greater willpower, grit and enthusiasm are experienced after which self-directedness develops where the self takes cognisance of what it can and cannot achieve alone. In this way, students are able to cooperatively collaborate with more abled others to achieve the academic goals. After all, it is the self that makes rational decisions and solves authentic problems. Therefore, within an AL environment, the self must possess a positive mind, attitude, values and knowledge in order to succeed (Gaidhu 2017).

Fifteen participants also stated that learners must be allowed to take risks to help them find themselves thus developing self skills. To be involved in meaningful learning, the self should have the freedom to try new things, reflect on them and then adjust the course of action if necessary to attain the desired goals whilst building self-confidence that supports a positive self-concept (Liao 2019). The gained self-confidence allows learners to take more academic risks and promotes a feeling of empowerment and emancipation. Thus, the self is afforded an opportunity to learn new things within a new, unfamiliar AL environment through engagement, exploration and enjoyment that ultimately leads to more self-development and self-knowledge. To encourage risk taking and acceptance of failure as part of meaningful learning within an unfamiliar but safe AL environment, learners can be encouraged to explicitly and continuously pose self-confidence enhancing questions such as How do I handle ambiguity and uncertainty? How do I view, feel and think about failure? (Winterman & Malacinski 2015). An emotionally and psychologically unsafe environment stifles the use and development of self skills, which in turn hampers the maturing of the learner. When learners fail to take interpersonal risks and accept failure, AL is limited resulting in inadequate and meaningless engagement and development of self where the learners fail to express themselves sensibly, rationally and openly (Kostovich, O’Rourke & Stephen 2020).

### Theme 2: Engage learners in activities that will consciously evoke authentic self

Nineteen participants stated that constantly thinking about self through engaging in self-awareness, self-dialogue, self-inquiry and self-reflection activities within an AL environment-assisted learners in evoking the authentic self. These activities can be embedded in real-life learning strategies such as community-based research and global exchange programmes. *Self-awareness* is the ability of a learner to assess a series of aspects regarding the self – such as perceptions, emotions, behaviours, attributes, goals and cognitive abilities (Muratore et al. 2019). By engaging in thought processes that assess own strengths, weaknesses, unspoken assumptions, biases and beliefs that influence the self when engaged in an AL environment, learners connect with their own conduct. They develop diverse self skills, therefore, become ‘self-ish’ as they learn more about themselves and their performance and as a result assume governance of the self (Formica 2008). ‘Self-ish’ skills such as self-insight, self-reflection, self-confident, self-motivation, self-accepting, self-empowerment, self-directedness, self-regulation and self-reliance help learners to understand others and be willing to empathise with them, which improves their interpersonal and professional relationships, thereby they develop self’s emotional intelligence. Self-aware individuals have higher-order thinking skills as they are able to make rational decisions and solve problems through critical thinking. They are capable of building good, therapeutic relationships with patients and their families across diverse, complex, dynamic healthcare settings and therefore are able to provide holistic, comprehensive and individualised care (Rasheed et al. 2020). The Johari window can be used as a reflective tool that can assist the self become consciously aware of those aspects pertaining to it, however, hidden from itself. Therefore, the learner can then appreciate that there are certain aspects of the self of which they might not be aware but that others can see. Such appreciation then requires the self to seek constructive feedback to build its awareness whilst taking cognisance of the fact that there will always be part of the self that is neither seen by self nor others, and those that are known to self but the self would rather disown (Jamieson & Davidson 2019). By knowing these aspects, a learner can be assisted in improving their learning and practices at a much deeper level and the quality of engagement with others including experts and peers while increasing their resilience (Morton, Jackson & Jackson 2020).

A lack of self-awareness leads to failure of realistically assessing oneself, whilst the deficits that one fails to see in oneself are occasionally apparent to others and, as a result, one is then seen as devoid of integrity. In addition to self-awareness, almost all participants also believed that learners must also engage in self-dialogue, self-inquiry and self-reflection.

*Self-dialogue* is a conversation that occurs within the self in which the self speaks, questions and answers itself (Nurbaity et al. 2018). Meaningful engagement in self-dialogue helps the learner to develop diverse self skills. They engage in the process of transformative learning in which the dialogical self allows itself to be a connection between the real world and itself, whilst gaining an understanding of these two entities (Jarvis 2018). However, nurse educators are warned that not every learner is able to constructively self-dialogue as most might actually engage in destructive, negative self-criticism (Nurbaity et al. 2018). Boulkraa (2016) suggested the use of a self-management cognitive-motivational strategy to enhance positive, constructive self-dialogue to promote learners’ attentional focus, perseverance, willingness, commitment, self-confidence, self-motivation and self-efficacy, all of which will assist them in attaining their academic goals. Furthermore, to produce an ingenious and outstanding self, nurse educators are advised to use optimistic self-talk (OST), realistic self-talk (RST) and ‘nowistic’ self-talk (NST). Constructive self-talk fosters a more active approach in solving authentic problems thereby enhancing cognitive self-control (Singh & Jain 2017). According to Slatyer et al. (2018), a mindful self-care and resiliency (MSCR) programme can provide learners with the ability to evade negative self-talk evoked by cognitive dissonance, ambiguity and uncertainty within an AL environment, and to consciously assume a more positive perspective on the situation as they become aware of their thought processes.

Negative self-talk is destructive in nature and may result in stress, anxiety, unproductivity, unsuccessful academic performance, and may hinder effective problem solving (Nurbaity et al. 2018; Singh & Jain 2017). It consumes the self and results in feelings of shame and guilt that impact upon self’s emotional wellbeing and as a result the self neglects its own emotional and psychological needs and thus becomes susceptible to compassion fatigue and burnout (Durkin et al. 2016). Within AL social groups such a community of learners, may perceive themselves as unworthy or socially undesirable (Brinthaupt et al. 2015), which may leave them unmotivated, and their academic growth and development stifled.

*Self-enquiry* involves questioning in the context of how the self thinks about itself (Malthouse, Watts & Roffey-Barentsen 2015). To facilitate self-awareness, learners must engage in self-questioning during their engagement in an AL environment. Such questions assist the self to further develop superior levels of tolerance for ambiguity and uncertainty inherent within an AL environment. A conscious engagement of learners in a supportive AL environment that will result in an experienced sense of cognitive dissonance will help them to explore different possibilities in pursuit of solving the authentic problems (Darling-Hammond et al. 2020). According to Frank and Stanszus (2019), to assist learners become future change agents with affective–motivational competencies, the self-inquiry-based learning (SIBL) and self-experience-based learning (SEBL) approaches may be employed. These approaches represent a holistic, experiential, action-oriented and transformational pedagogy that supports self-directed and problem-oriented learning.

*Self-reflection* is ‘the inspection and evaluation of one’s thoughts, feelings and behavior’ (Chen, Chen & Pai 2019:63), which involves a process of self-analysis, self-evaluation, self-dialogue and self-observation (Toros & Medar 2015). The continuous use of self-reflection deepens the understanding of the AL environment and improves the self’s personal and professional awareness, which may result in the positive therapeutic use of self in practice. Such awareness is improved through continual assessment of everyday practices and personal belief systems. However, self-reflection needs to be deeper to develop stronger self-awareness and good critical thinking skills (Hwang et al. 2018). These critical thinking skills are defined as ‘self-directed, self-disciplined, self-monitored and self-corrective thinking’ by Paul and Elder (The Foundation for Critical Thinking 2019:2). This implies that self-reflection facilitates self-awareness and provides the self with opportunities for self-emancipation. To assist learners in developing constructive self-reflection, particularly when dealing with experiences of unnecessary guilt or self-criticism, critical reflective journaling can be used (Hwang et al. 2018). According to Walker and Lovat (2015), a non-reflective self may lose itself and as a result may experience undesirable symptoms such as burn-out, disconnectedness and a feeling of insignificance, which may lead to engagement in unintentional, life-threatening practice to the patient. An example of such practice may include a non-reflective self unintentionally disconnecting a pacemaker from which a critically ill patient might be highly dependent as the self might not be reflecting-in-action about what that it is doing and why. The learners are then inclined to be descriptive in their thinking, following routine to the letter and are highly dependent on empirical-analytical knowledge.

Nineteen participants stated that well-developed self skills in a learner develop an *authentic self*. According to Chinn and Kramer (2015:115), personal knowing is the ‘basis for expression of authenticity, the genuine self, which in turn is essential in a healing relationship’. To develop authentic self, learners need to develop self-awareness as well as know how others perceive the self since honest self-awareness assists the self in making choices that are intentional, thus, align with the real, genuine, true self. This results in a more expansive identity and enhances creativity as well as reliance on intuition (Schofield et al. 2013). The authentic self in learners may be developed through adoption of an innovative, learner-centred problem-solving heuristic approach that is self-inviting and supports self-directed growth, development and advancement, whilst utilising the learners’ real-life experiences and prior knowledge to develop their problem-solving and discovery skills (Ofori-Kusi 2017). The views of these authors support the views of the participants in this study.

### Theme 3: Evaluation of developed self skills and metacognition

According to the participants, metacognition develops from well-developed self skills, and whilst all skills must be taught, they must also be modelled and evaluated. Similarly, according to Fauzi and Sa’diyah (2019), well-developed self skills develop metacognition as well as assertiveness, self-insight and emotional intelligence. Metacognition is defined as the ‘awareness of one’s own thinking and capacity for strategic action’ (Pakdaman-Savoji, Nesbit & Gajdamaschko 2019:5). However, for the self to develop metacognitive skills, metacognitive knowledge as a prerequisite for and an outcome of metacognitive skills is required. With developed metacognitive skills, the self is not only enabled to effectively and efficiently solve authentic problems through identification of current knowledge, skill and attitude, planning, monitoring, evaluation and allocation of resources with optimal efficiency (Gholami et al. 2016) but also to assess such problems from different perspectives and as a result find innovative multifaceted solutions (Jumari et al. 2018). The self thus has more organised thoughts and uses metacognitive knowledge and regulation to interpret newly acquired knowledge to improve their academic performance. Learners are enabled to recognise their flaws and develop and effectively manage their new cognitive skills. According to Gholami et al. (2016), poor metacognitive skills render the self unable to think critically, solve authentic problems and integrate theory into practice. The self then becomes indecisive with poor clinical reasoning resulting in poor clinical practice and is unlikely to succeed academically as it does not use metacognitive strategies to overcome deficits (Kosior, Wall & Ferrero 2019).

### Strengths and limitations

In spite of the limitations mentioned below, the findings proved to be relevant, robust and provided rich description on how learners’ self skills can be developed by nurse educators within an AL environment. The study adopted a qualitative research design to answer the research question using a limited number of participants in a higher education institution, which limits the generalisability of the findings. The study, however, could be replicated in similar contexts by other interested researchers. Only the views of nurse educators were explored and described, and not those of learners.

## Implications and recommendations

### Nursing practice and research

Whilst self skills are imperative in an AL environment during training as well as in healthcare settings, knowing self is not without its pitfalls. The process of knowing self can conditionally contribute to increased stress and depressive symptoms (Nakajima, Takano & Tanno 2018). It is thus recommended that the views of learners on how their self skills can be developed within an AL environment be explored and examined against those of nurse educators and that specific recommendations be made. Future quantitative studies should be conducted to evaluate the developed self skills using the mentioned tools and questionnaires. Furthermore, the effectiveness of the developed self skills in the provision of quality nursing care during nursing practice needs to be determined.

### Nursing education

Nurse educators should not attempt to facilitate self skills if they themselves lack such skills. For a nurse educator to be able to facilitate self-awareness, they need to be self-aware (Ndawo 2017). It is recommended that nurse educators use the pedagogy of vulnerability in which ‘they render their frames of knowing, feeling and doing vulnerable’ through critical self-dialogue and self-disclosure to assist learners develop their authentic self (Brantmeier 2013). They also need to develop their own metacognitive practices as well by asking self-analytical and reflective questions related to themselves and their facilitation of development of self skills in learners within an AL environment. These metacognitive practices will assist nurse educators develop metacognition for their learners. Furthermore, they should attend workshops on metacognitive instruction for teachers and use adaptive metacognition techniques that explicitly make them aware of their metacognition to facilitate effective teaching practice that will benefit the learners.

The development of self skills and metacognition is challenging and time consuming as these skills develop, grow and improve over time. However, once developed, the self is enabled. It is thus recommended that nurse educators exercise patience and perseverance to assist learners develop these skills properly because the more the learners use these self skills, the more readily they will develop them (Jumari et al. 2018). It is further recommended that nurse educators make the teaching of self skills compulsory in the curriculum within an AL environment with explicitly stipulated objectives and teaching, demonstration and evaluation strategies, the same way cognitive and psychomotor skills are taught (Benn 2018; Kosior et al. 2019; Yu, Ling & Hu 2019).

## Conclusion

The self makes rational decisions and finds effective solutions for authentic problems. The findings in this study indicate that the development and use of self skills may be beneficial to individual learners and communities across a range of domains. Nursing education should empower learners with good self skills that will help them to adapt to the rapidly changing healthcare environment (Driscoll 2016). Knowing self in an AL environment is important as such knowledge impacts on the way a learner interacts and engages with significant others in a real-world working environment. Whilst the self is expected to deliver quality patient care and develop solutions to global complex problems, it also continues to handle cognitive complexities and conditions in which there is little knowledge whilst creating its own identity. If one has a healthy sense of self, the quality nursing care that one delivers will be altruistic, comprehensive, holistic and individualised. At the same time, the nursing will be infused with wisdom, love and compassion (Benn 2018). This study has led to a deeper understanding of nurse educators’ views on how self skills can be developed in learners within an AL environment. It also contributes to the body of knowledge by supporting the view that self skills and their development in learners are vital for effective and compassionate nursing.
